# Complex distribution of EFL and EF-1α proteins in the green algal lineage

**DOI:** 10.1186/1471-2148-7-82

**Published:** 2007-05-23

**Authors:** Geoffrey P Noble, Matthew B Rogers, Patrick J Keeling

**Affiliations:** 1 Canadian Institute for Advanced Research, Department of Botany, University of British Columbia, 3529-6270 University Boulevard, Vancouver, BC V6T 1Z4, Canada

## Abstract

**Background:**

EFL (or elongation factor-like) is a member of the translation superfamily of GTPase proteins. It is restricted to eukaryotes, where it is found in a punctate distribution that is almost mutually exclusive with elongation factor-1 alpha (EF-1α). EF-1α is a core translation factor previously thought to be essential in eukaryotes, so its relationship to EFL has prompted the suggestion that EFL has spread by horizontal or lateral gene transfer (HGT or LGT) and replaced EF-1α multiple times. Among green algae, trebouxiophyceans and chlorophyceans have EFL, but the ulvophycean *Acetabularia *and the sister group to green algae, land plants, have EF-1α. This distribution singles out green algae as a particularly promising group to understand the origin of EFL and the effects of its presence on EF-1α.

**Results:**

We have sampled all major lineages of green algae for both EFL and EF-1α. EFL is unexpectedly broad in its distribution, being found in all green algal lineages (chlorophyceans, trebouxiophyceans, ulvophyceans, prasinophyceans, and mesostigmatophyceans), except charophyceans and the genus *Acetabularia*. The presence of EFL in the genus *Mesostigma *and EF-1α in *Acetabularia *are of particular interest, since the opposite is true of all their closest relatives. The phylogeny of EFL is poorly resolved, but the *Acetabularia *EF-1α is clearly related to homologues from land plants and charophyceans, demonstrating that EF-1α was present in the common ancestor of the green lineage.

**Conclusion:**

The distribution of EFL and EF-1α in the green lineage is not consistent with the phylogeny of the organisms, indicating a complex history of both genes. Overall, we suggest that after the introduction of EFL (in the ancestor of green algae or earlier), both genes co-existed in green algal genomes for some time before one or the other was lost on multiple occasions.

## Background

Horizontal or lateral gene transfer (HGT or LGT) is the non-sexual movement of genetic information between two species. This process has been amply documented to affect prokaryotic genomes: although the frequency and importance of such events is debated, there is little debate that it plays some role [1-4]. The importance of HGT in the evolution of eukaryotic genomes is not as well appreciated, in part because comparative nuclear genomics has lagged behind that of prokaryotes. Nevertheless, a number of genome-wide surveys have now shown the process has had a broad impact in several species [5-9], and a number of single gene transfers have also been investigated and found to have interesting implications for ecology, protein function, or genome evolution [10-12].

One potential gene transfer with interesting functional implications is EFL, or elongation factor-like protein [13]. EFL is member of a large GTPase superfamily containing translation initiation, elongation, and termination factors. EFL is specifically related to a subset of proteins found exclusively in eukaryotes, including elongation factor-1 alpha (EF-1α), eukaryotic translation release factor-3, and HSP70-binding protein HBS1. EFL is itself only known from eukaryotes, but not all eukaryotes have it. It has presently been reported from eight eukaryotic lineages [13-15], nearly all of which have close relatives which lack it (many of which have completely sequenced genomes supporting the absence of EFL). Moreover, most or all the lineages where EFL has been reported to date appear to lack EF-1α (again, this includes both organisms with completely sequenced genomes and some with large scale EST sequencing surveys). Altogether there is generally a mutually exclusive distribution of EFL and EF-1α, and both are scattered around the tree of eukaryotes. This is of interest for several reasons. First, EF-1α was formerly considered essential because of the important and universal role it plays in translation, but this is clearly not the case since some EFL containing organisms clearly don't use EF-1α. Second, the distribution suggests EFL might substitute or partially substitute for EF-1α function. Third, the punctate distribution of EFL suggests it may be moving between eukaryotes [13]. As more lineages with EFL are found [14,15], the alternative that EFL is ancestral to all the lineages that possess it, and that either EFL or EF-1α has been lost many times in different lineages becomes more defensible. However, the number of such events is very large since the lineages possessing EFL are scattered across the tree of eukaryotes, and the number of lineages with EFL remains far fewer than those with EF-1α. Based on the current distribution we favour the movement of EFL between eukaryotes, but this must be reassessed as the distribution becomes better known.

Within each group where EFL has been found, the distribution of the protein is sometimes simple but uninformative; for example, all dinoflagellates appear to use EFL[14]. Conversely, it's distribution among other groups is more complex but difficult to interpret; for example, some fungi, ichthyosporeans, and choanoflagellates have EFL while others do not and the relationships between these is unclear [13,15-17]. A group where the distribution appeared to be most precisely explained was the green algae: here the well-sampled plants lack EFL and instead have EF-1α, whereas the well-sampled chlorophyceans and trebouxiophyceans have EFL and lack EF-1α. The single ulvophycean eukaryotic elongation factor, from *Acetabularia acetabulum*, was found to use EF-1α but not EFL, altogether leading to the conclusion that the green algal EFL originated in a common ancestor of chlorophyceans and trebouxiophyceans {Keeling, 2004 #652}. This makes green algae a good model for the study of the fine scale distribution of EFL for two reasons. First, EFL seems to have arisen within the group rather then prior to its diversification, so the age of this event could in principle be predicted. Second, while the phylogeny of green algae is not perfectly known, it is better understood than most other eukaryotic groups [18], so the distribution can be interpreted according to a reasonable phylogeny. A better idea of this distribution can help us answer several questions. In particular, if EFL is introduced into a lineage by HGT, what happens to EF-1α? Do they both persist for some time, and does lineage sorting take place? Only a fine-scale survey of a group where EFL exists in some but not all members could address such questions, and these questions are important because they are one way to determine the possible functional relationship between EFL and EF-1α.

Here we have sampled EFL and EF-1α from representatives of all major missing lineages of green algae. The viridiplantae are divided into two main groups, streptophytes and chlorophytes [18]. The streptophytes include land plants, charophyceans, and the enigmatic scaly green flagellate *Mesostigma*. The chlorophytes include four subdivisions, prasinophyceans, ulvophyceans, trebouxiophyceans and chlorophyceans (we will use the suffix '-ceans' so as to distinguish the larger group chlorophytes from the subgroup chlorophyceans). The expectation, based on the relatively simple distribution originally described, is that EF-1α but not EFL should be found in all green lineages except chlorophyceans and trebouxiophyceans. In contrast, however, we have found that EFL is abundant in chlorophytes and EF-1α comparatively rare. Indeed, among chlorophyte green algae, *Acetabularia *is the only lineage in which we could find EF-1α: EFL was found in all other chlorophyte lineages, and in the streptophyte *Mesostigma*. Phylogenetic reconstruction shows the *Acetabularia *EF-1α is related to homologues from charophytes and land plants, and therefore ancestral (and the only known chlorophyte EF-1α to be retained). This implies that EFL and EF-1α were likely both present in the ancestor of streptophytes and chlorophytes, and that lineage sorting led to the current distribution of EFL and EF-1α. This has implications for the functional co-existence of the two proteins, and also suggests that EFL was more frequently retained than was EF-1α.

## Results & discussion

### Characterisation of new EFL and EF-1α genes from green algae

Previously, EFL was found in a number of species of chlorophyceans and trebouxiophyceans, but not in any land plants or the ulvophycean *A. acetabulum*. This lead to the conclusion that it originated in a common ancestor of chlorophyceans and trebouxiophyceans [13], which in turn leads to the prediction that all other green algae should only contain EF-1α. In contrast, however, we have found that most green algal lineages possess EFL. Genes encoding EFL were characterized from three prasinophyceans (*M. pusilla*, *T. tetrathele*, and *O. tauri*), two ulvophyceans (*U. fenestrata *and *U. intestinalis*), an additional chlorophycean (*Chlorococcum *sp.), and the only mesostigmatophycean (*M. viride*). From none of these lineages were we able to detect the presence of a gene for EF-1α. Moreover, EF-1α was not present in the complete genome of *O. tauri *or in EST surveys of *M. viride *[19,20]. An EST survey of a third ulvophycean, *U. linza *[21], was also searched and in agreement with our results contained a short fragment of EFL but no detectable EF-1α. Conversely, EF-1α was characterized from two charophyceans (*C. australis *and *Spirogyra *sp.), and we were not able to detect the presence of EFL in either of these species. EFL fragments were identified in EST or genomic survey projects from four land plants, *Pinus taeda*, *Oryza sativa*, *Lactuca saligna*, and *Triticum aestivum*. With the exception of the first two, however, these do not form a group in phylogenetic analyses (Suppl. Figure 1) as one would expect if they were ancestrally land plant sequences. Moreover, most are closely related to either a subset of green algae or fungi, and are poorly represented in the surveys where they are found. In addition, in cases where substantial genomic data are available from the same organism, the EFL is only found in EST sequences and not in the genomic data. The best cases are *O. sativa *and *T. aestivum*, where Blast searches against their respective genome databases revealed no EFL sequence, and specifically no match to the EST purportedly from that organism. On balance, we conclude that these are contaminant sequences until evidence that they are truly encoded in the plant genomes is available (which, according to the phylogeny, would suggest several recent acquisitions in certain plants).

**Figure 1:**
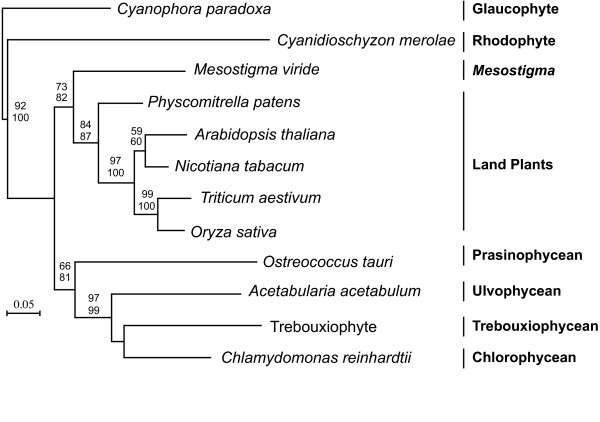
**Bayesian phylogeny of concatenated proteins from green algae and plants with maximum likelihood branch lengths**. Numbers at nodes correspond to bootstrap support from protein maximum likelihood methods ProML (top) and PhyML (bottom). Major groups are labeled to the right. Note that the relationships between *M. viride *and streptophytes and *A. acetabulum *and chlorophytes are both recovered and supported by bootstrap. The 'Trebouxiophyte' is a composite of sequences from *Chlorella*, *Prototheca*, and *Helicosporidium *(for which genes come from each taxon, see methods).

In general, organisms from the streptophyte lineage mostly possess EF-1α while organisms from the chlorophyte lineage mostly possess EFL. However, *M. viride *and *A. acetabulum *are important exceptions because, based on their currently accepted taxonomic positions, one would predict that they should use EF-1α and EFL respectively, whereas EST sampling from both reveals the opposite. It is possible that both genes are present in these genomes, although this would still suggest the expected gene is not expressed as highly as the one characterized here, especially in *M. viride*, where there is a large sample of 10,395 ESTs [19]. It is also possible that these species are misclassified; that *M. viride *is really a chlorophyte and *A. acetabulum *is really a streptophyte. The phylogenetic position of both genera, and in particular *M. viride*, have been analysed many times using a variety of data [18,19,22-27], making this somewhat unlikely. Nevertheless, we carried out a phylogeny based on nine concatenated protein-coding genes from the EST surveys to re-confirm their phylogenetic affinities to streptophytes or chlorophytes. The phylogeny (Figure 1) is consistent with previous analyses that place *M. viride *at the base of the streptophytes and *A. acetabulum *within the chlorophytes. This rules out the possibility that the possession of EF-1α and EFL by *A. acetabulum *and *M. viride*, respectively, reflects their relationship to other groups that possess the same gene. In short, the distribution of EFL and EF-1α is not strictly consistent with the phylogenetic relationships of the organisms where the proteins are found.

### Phylogenies of EFL and EF-1α

The complex distribution of EFL and EF-1α could have arisen from multiple transfers of EFL to green algae, reversions by re-introduction of EF-1α, or an extended period of time where both genes co-existed in the same ancestral lineage followed by differential loss in the descendants of this lineage. Each of these hypotheses is significant for different reasons, and each leads to predictions for the phylogeny of EFL and EF-1α. If EFL invaded green algae multiple times, the green algal EFLs will not form a single well-supported clade. If EF-1α was re-introduced to one or more groups, then the green algal and plant EF-1α genes will not form a single well-supported clade. Lastly, if lineage sorting has taken place, then the green lineage should form a single clade in both genes.

**Figure 2:**
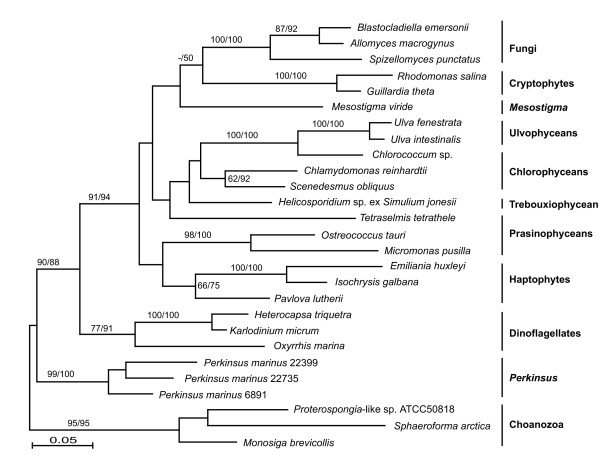
**Bayesian phylogeny of EFL proteins with maximum likelihood branch lengths**. Numbers at nodes correspond to bootstrap support from protein maximum likelihood methods ProML (left) and PhyML (right). Major groups are labeled to the right. Note that the relationships of many of the green algal groups are not well supported, and that they fall into three poorly separated groups.

The first possibility cannot be confidently accepted or rejected because the phylogeny of green algal EFL genes is not sufficiently well resolved. Figure 2 shows a phylogeny of all full-length EFL genes with the exception of the highly divergent gene from the chlorarachniophyte *Bigelowiella natans*; this gene has been included in analyses previously [13], and we similarly found no indication that it was related to green algal EFL (Suppl. Figure 1). The green algal EFL sequences fall into three groups. One large and weakly supported clade includes representatives of all four major chlorophyte subgroups (noteworthy among which are the ulvophyceans since *Acetabularia *is currently believed to be related to this group). A second strongly-supported clade includes marine picoplanktonic prasinophyceans (noteworthy among which is a previously unidentified environmental sequence from the Sargasso Sea that is specifically related to *M. pusilla*, consistent with the source of this material and the size of cells collected: Suppl. Figure 1). Lastly, *M. viride *branches independently of other green algae without any support for its position, which is unfortunate since it would be expected to possess EF-1α rather than EFL. None of these three groups of green algae is related to any other group or to one another with strong support in any analysis.

**Figure 3:**
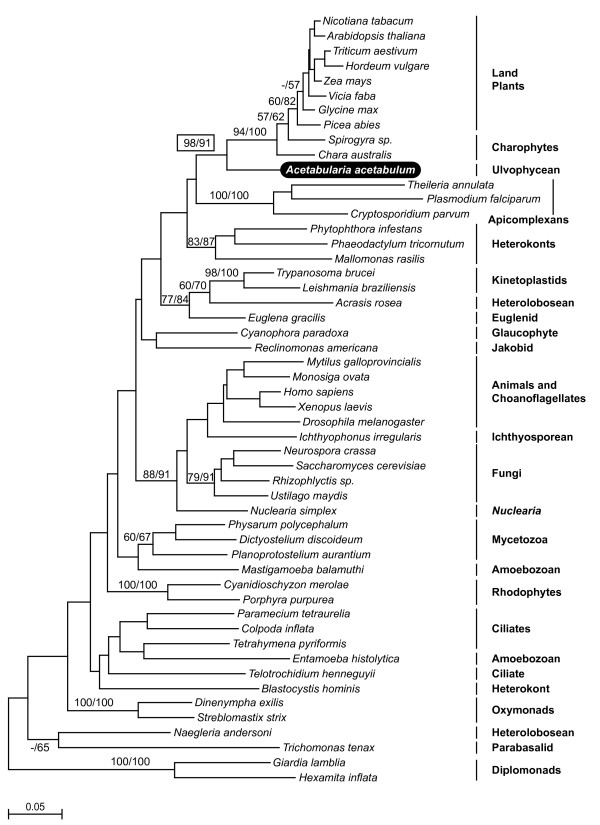
**Bayesian phylogeny of EF-1α proteins with maximum likelihood branch lengths**. Numbers at nodes correspond to bootstrap support from protein maximum likelihood methods ProML (left) and PhyML (right). Major groups are labeled to the right. Note that *A. acetabulum *is sister to the streptophytes and that the charophyceans are sister to plants, in agreement with previous molecular phylogenies of the viridiplantae.

The phylogeny of EF-1α is much more informative (Figure 3). In contrast to EFL, the positions of all members of the green lineage, *A. acetabulum*, charophyceans, and land plants, are resolved with strong support, and the relationships of the EF-1α genes match those expected for the organisms. The green clade is well supported overall (91–98%), with *A. acetabulum *sister to all streptophytes with 94–100% support. The charophyceans are paraphyletic with *Spirogyra *sister to land plants, although this branching order is not supported by bootstrap analyses. The position of *A. acetabulum *is of particular importance, because the presence of EF-1α is unexpected in this ulvophycean, given chlorophytes as a whole, including other ulvophyceans, do not appear to possess EF-1α. In previous analyses based on a truncated EST the position of the *A. acetabulum *EF-1α was not resolved with strong support, but it did not branch with plants [13]. However, having now sequenced an additional 561 bp of the 5' end (about 50% of the alignable sequence), it is clear that the *A. acetabulum *EF-1α is indeed sister to other members of the green lineage. This suggests very strongly that *A. acetabulum *has not 'reacquired' EF-1α secondarily (e.g., through HGT), and by extension that its EF-1α gene is the single known representative of the ancestral chlorophyte EF-1α.

### The evolution and distribution of EFL in the green algal lineage

**Figure 4:**
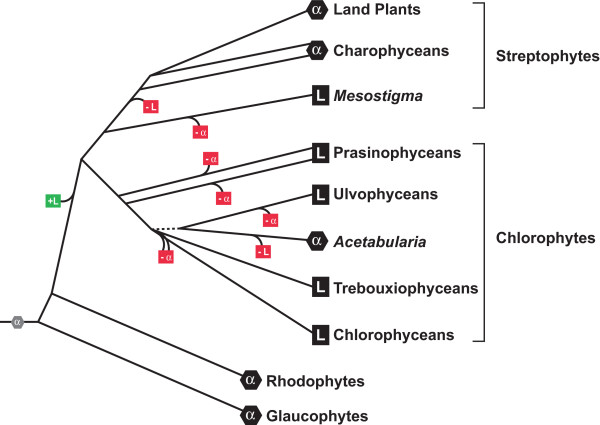
**Schematic of evolutionary relationships within the green algal lineage showing distribution of EFL and EF-1α and possible events explaining the distribution**. A phylogeny of the Plantae, with the known presence of either EFL or EF-1α in extant lineages plotted at the tips of each branch (EFL is a square containing "L" and EF-1α is an octagon containing an "α"). All known rhodophytes and glaucophytes contain EF-1α, as do most other eukaryotic lineages, so the ancestor in inferred to have contained EF-1α as well (indicated by a gray octagon at the base of the tree). Also plotted is one possible explanation for the current distribution of the two genes in the green lineage, where EFL was gained once in the ancestor of the green viridiplantae (indicated by the green box), and subsequently either EFL or EF-1α lost in several lineages each (indicated by red boxes with indicated which gene would have been lost). Other models to explain this distribution that include multiple origins of EFL are also possible, but are not shown for simplicity. The tree of the green lineage is based on Figure 1 and other analyses [18].

The range of green algae that possess EFL is much broader than expected based on previous data, and more importantly its distribution is not easily reconciled with the evolutionary relationships among the organisms that possess it. On the whole, streptophytes possess EF-1α, with the exception of *M. viride*, whereas chlorophytes possess EFL, with the exception of *A. acetabulum*. Figure 4 shows a schematic phylogeny of the green lineage with the distribution of EFL and EF-1α. The phylogeny of EF-1α demonstrates that EF-1α existed in the ancestor of the green lineage, and that the land plants and charophyceans as well as the chlorophyte *A. acetabulum *have all retained this ancestral gene.

It is possible that the distribution of EFL is due to multiple independent invasions of the gene to different subgroups of the green lineage. This would require three or perhaps more origins of EFL (this is unclear because the phylogeny of basal chlorophytes is not resolved), which would suggest that EFL was very mobile indeed. While this is consistent with the present data (because the EFL tree does not reject such a scenario), it seems unlikely that one group would acquire such a gene many times.

Another possibility is that EFL and EF-1α were both present in the common ancestor of streptophytes and chlorophytes and co-existed for some extended period of time. Assuming that both genes do not still co-exist in any one green algal genome, this would mean EFL was lost once in the ancestor of charophyceans and land plants, and once in an ancestor of *A. acetabulum*, whereas EF-1α was lost once in an ancestor of *M. viride *and several times in chlorophyte lineages. Because the phylogeny of chlorophytes is still uncertain it is impossible to state how many times EF-1α would have to have been lost, but it is interesting that EF-1α loss would likely have been more frequent than EFL loss. It is also possible that both genes persist in some green algae, although we know this is not the case in several plants, *O. tauri*, and *C. reinhardtii*, and have good reason to suspect it is not true in the many green algae for which there are large EST surveys. A long period of co-existence followed by differential loss of one or the other gene may seem unlikely at first glance, but if one considers the function of these proteins it may not be so. More specifically, EFL has been argued to share some functional overlap with EF-1α in translation [13]. However, EF-1α is a multifunctional protein, so even if EFL is capable of taking over its role in translation elongation, it stands to reason that EF-1α would have to persist until its other roles in the cell were also obsolete or at least non-essential. Accordingly, a period of co-existence is probably not only possible, but necessary, and during such a time either protein could conceivably be lost. This has the interesting implication that plants, although they now only have EF-1α, once had EFL as well. It also bolsters expectations that organisms with both proteins should exist. The zygomycete fungus *Spizellomyces punctatus *is reported to have both genes [17], and we have detected both in the diatom *Thalassiosira pseudonana *(G. Gile and PJK, unpublished data), but so far co-existence of the two proteins is curiously rare.

## Conclusion

We have shown that the apparently mutually exclusive distribution of the GTPase genes EFL and EF-1α in the green lineage is not consistent with the phylogeny of the organisms in which the genes are found. Specifically, members of the streptophyte lineage tend to encode EF-1α and not EFL, except for the basal genus *Mesostigma *where the opposite is found. Conversely, members of the chlorophytes tend to encode EFL and not EF-1α, except for *Acetabularia *where the opposite is found. The phylogeny of EFL does not resolve the origin of the *Mesostigma *gene, but the phylogeny of EF-1α clearly shows the *Acetabularia *gene to be a relict, ancestral chlorophyte EF-1α rather than an independent re-acquisition. Altogether, we suggest that the ancestor of the green lineage encoded EF-1α, but at some point EFL was introduced and the two genes co-existed in green algal genomes for some time before one or the other was lost on multiple occasions.

## Methods

### Strains

Multiple whole plants of *Ulva fenestrata *and *Ulva intestinalis *were collected from the intertidal zone at Spanish Banks, Vancouver, BC Canada. *Micromonas pusilla *(strain NEPCC 29), *Chlorococcum *sp. (strain NEPCC 478), *Tetraselmis tetrathele *(strain NEPCC 500) were grown in natural seawater medium [28] at 16° with a 16:8 light:dark cycle. *Chara australis *(strain FWAC 7144) and *Spirogyra *sp. (strain FWAC 125) were grown in soilwater medium [29]at 16° with a 16:8 light:dark cycle. A cDNA library from *Acetabularia acetabulum *(strain Aa0005) was generously provided by D. Mandoli.

### Characterisation of EFL and EF-1α genes

Material from all cultures was harvested by centrifugation and total RNA and DNA was isolated using protocols described previously [30]. RNA and DNA was used as a template for RT-PCR and PCR reactions, respectively, using primers for each of EFL and EF-1α. EFL primers were CTGTCGATCGTCATHTGYGGNCAYGTNGA and CTTGATRTTNAGNCCNACRTTRTCNCC, EF-1α primers were AACATCGTCGTGATHGGNCAYGTNGA and CTTGATCACNCCNACNGCNACNGT or CAACATCGTCGTCATCGGNCAYGTNGA and GCCGCGCACGTTGAANCCNACRTTRTC. Products of the expected size (or larger in the case of genomic DNA amplifications) were cloned and multiple clones completely sequenced. In all cases the RT-PCR approach was more successful, leading us to conclude EFL and EF-1α may encode a number of introns. The *A. acetabulum *EFL was partially characterized as part of an EST survey [13], and to improve the resolution of its phylogenetic position we characterized the 5' portion of the gene by amplification from a cDNA library using the specific primer TTCCGACCGGCACGGTTCCAATTCCG and the 5' degenerate EF-1α primer AACATCGTCGTGATHGGNCAYGTNGA. During the course of this work the complete nuclear genome of the prasinophycean *Ostreococcus tauri *and an EST survey from the mesostigmatophycean *Mesostigma viride *(strain NIES 476) both became available [19,20]. EFL and EF-1α were both sought from this sequence data, from which only EFL sequences could be found. New sequences were deposited in GenBank under accession numbers EF551321-EF551331.

### Phylogenetic analyses

New EFL and EF-1α sequences were added to existing amino acid alignments [14]. The two proteins were analysed separately since their relationship to one another has been examined previously, and separate analyses allow the inclusion of more unambiguously alignable characters, 425 and 407 for EFL and EF-1α, respectively. An alignment of nine concatenated proteins from algae and plants was also constructed using actin, alpha-tubulin, beta-tubulin, RbcS, Rps10, Rps13, Rpl3, Rpl11, and Rpl13, for a total of 1,206 unambiguously alignable amino acid characters (with no missing data). The trebouxiophytes are represented by a composite of sequences from three species: actin, Rps10, and Rpl11 are from *Helicosporidium *sp., beta-tubulin, Rpl3, Rpl13 and Rps13 are from *Prototheca wickerhamii*, and alpha-tubulin, RbcS, and TufA are from *Chlorella vulgaris*. Trees were inferred using distance, maximum likelihood and Bayesian methods. Bayesian trees were inferred using Mr. Bayes 3.1 [31] employing the WAG substitution model with site-to-site rate variation modeled on a gamma distribution with 8 variable rate categories and one category of invariable sites, three heated chains and one cold one, and 1,000,000 generations with sampling every 1,000 generations. Log likelihoods were plotted and showed a rapid plateau after only five samples, so a burnin of 40 trees was removed before constructing the consensus (constructing a consensus of all trees resulted in the same topology). Maximum likelihood branch lengths for the consensus topology were calculated using ProML 3.6 [32] with JTT, 8 gamma rate categories and one category of invariable sites. ProML trees were inferred using the same settings, and 100 bootstraps were inferred with the gamma shape parameter alpha and proportion of invariant sites estimated using Tree-Puzzle 5.2 [33]. ML trees and 1,000 bootstrap trees were also inferred using PhyML 2.4.4 [34] with the WAG model with 8 gamma rate categories and one category of invariable sites (when p-inv was not zero), and parameters estimated from the data. Distances were also calculated using Tree-Puzzle with the same settings (and parameters estimated from the data), and trees constructed using WEIGHBOUR 1.0.1a [35]. 100 bootstrap replicates were carried out using puzzleboot [36], which gave similar results as maximum likelihood (not shown).

## Authors' contributions

G.P. Noble and P.J. Keeling characterized all new gene sequences. Phylogenetic analyses were performed by M.B. Rogers, G.P. Noble and P.J. Keeling. P.J. Keeling conceived of the study and wrote the manuscript.

## Supplementary Material

Additional file 1 Bayesian phylogeny of all known EFL genes and fragments with maximum likelihood branch lengths. Numbers at nodes correspond to bootstrap support from protein maximum likelihood methods ProML (top) and PhyML (bottom). Major groups are labeled to the right. Click here for file
